# Treatment outcomes of patients with primary tracheal tumors - analysis of a large retrospective series

**DOI:** 10.1186/s12885-024-12450-z

**Published:** 2024-06-05

**Authors:** Aleksandra Piórek, Adam Płużański, Magdalena Knetki-Wróblewska, Kinga Winiarczyk, Sylwia Tabor, Paweł Teterycz, Dariusz M. Kowalski, Maciej Krzakowski

**Affiliations:** 1 Department of Lung Cancer and Thoracic Tumors, Maria Sklodowska-Curie National Research Institute of Oncology, Roentgena 5, Warsaw, 02-781 Poland; 2 Department of Soft Tissue/Bone Sarcoma and Melanoma, Maria Sklodowska-Curie National Research Institute of Oncology, Warsaw, 02-781 Poland; 3 Department of Computational Oncology, Maria Sklodowska-Curie National Research Institute of Oncology, Warsaw, 02-781 Poland

**Keywords:** Primary tracheal cancer, Adenoid cystic carcinoma, Squamous-cell carcinoma of the trachea, Treatment outcome, Survival

## Abstract

**Objective:**

Primary tracheal tumors are very rare and their management is not definitely established. Due to its rarity, providing patient care in terms of optimal management poses a considerable challenge. The purpose of this study was to investigate treatment outcomes in patients with these rare tumors.

**Methods:**

We carried out a retrospective analysis of 89 patients with primary tracheal tumors treated at the Maria Sklodowska-Curie National Research Institute of Oncology in Warsaw, Poland, over sixteen years. The study assessed patient demographics, tumor characteristics and treatment. Different treatment options were compared in terms of overall survival, disease-free survival, and progression-free survival.

**Results:**

A total of 89 patients were included in the study. In the group presented, 45 patients underwent primary radical treatment and 44 were qualified for palliative treatment. Surgical resection was performed in 13 patients out of radically treated patients. The 5 year OS rates in the group of patients who underwent radical treatment and in the group of patients who underwent palliative treatment were 45.9% and 2.3%, respectively. In the group of patients who underwent radical surgical treatment, the 5 year OS was 76.9% compared to 35.8% in the group of patients who underwent nonsurgical treatment.

**Conclusion:**

A multidisciplinary team should decide treatment options, including in-depth consideration of surgical treatment options.

## Introduction

Primary tracheal tumors are rare and their management is not definitely established. They represent 0.2% of all respiratory cancers and 0.02–0.04% of all malignancies [1], with an annual incidence of approximately 0.1 per 100,000 people [2–5]. The most common types are squamous-cell carcinoma (SCC) and adenoid cystic carcinoma (ACC), which together account for more than two thirds of primary tracheal tumors in adults [6]. The prognosis of patients with primary tracheal tumors is determined by several factors. Histological diagnosis of ACC [3, 7–19], better performance status [14, 16, 20–22], and radical surgery [3, 7, 8, 16, 18, 23–26] have been identified as favorable prognostic factors. Our knowledge about the primary tracheal tumors is limited mainly to reviews of retrospective analyses and case series descriptions. Characteristics of the various histologic types of cancers have been similar across the reviews, but tumor management has varied widely.

In this retrospective study, we investigated treatment outcomes according to treatment modalities in patients with primary tracheal tumors. We examined our institutional experience and compared it with that reported previously.

## Materials and methods

This retrospective analysis included patients with primary tracheal tumors treated at the National Research Institute of Oncology in Warsaw, Poland, between January 2000 and December 2016. All patients were included in our previously reported analysis describing the TNM (tumor, node, metastases) staging system and the prognostic significance of sex in patients with primary tracheal tumors [27, 28]. The patients were identified by searching the institution’s cancer registry. We enrolled adults (≥ 18 years) diagnosed with primary tracheal tumors. Patients with tumors that may have originated in the larynx, the main bronchus, or other organs (e.g., thyroid or esophagus) were excluded. Because tracheal neoplasms are not included in the International Union Against Cancer (UICC) and American Joint Committee on Cancer (AJCC) classification systems, staging was performed retrospectively on the basis of available imaging results before qualification for treatment. The location and the extent of the disease were estimated from the baseline computed tomography scans and descriptions of bronchoscopic examinations if available. Positron emission tomography–computed tomography examination was only available in individual cases.

In general, the records of 89 actively treated patients with primary tracheal tumor were included. Data on demographics, clinicopathological variables and type of treatment were extracted from medical records. The prognostic value of selected clinical and morphological factors was evaluated. Due to the heterogeneity of the study population, analyses were performed for the entire group and for subgroups, depending on the intention of treatment (i.e. radical or palliative). Survival information was obtained from medical records and from the offices keeping records of population movement. The patients were presumed dead if their name, date of birth, and PESEL (Universal Electronic System for Registration of the Population) number matched. The median follow-up was 93.4 months (95% CI: 76.4–NR). Follow-up examinations included a computed tomography scan every 3 months for the first year and then every 6 months. Bronchoscopy was performed when necessary. The overall survival (OS) was calculated from the date of the first diagnosis until death from any cause. The study was carried out according to the Declaration of Helsinki and the Institutional Review Board of the National Institute of Oncology Research Institute (opinion number 20/2019, January 16, 2019).

Patient demographics, tumor characteristics, and details of treatment and tumor response were summarized using the number of patients and percentages of the whole group. Differences between groups were evaluated using the Mann–Whitney U test. The Kaplan–Meier method for estimating survival functions and the Cox proportional hazards model for estimating the effects of covariates on the hazard of the occurrence of death were used. All confidence intervals (CI) were 95%. All *p*-values < 0.05 were considered significant. No adjustment was made for multiple tests. All analyses were performed in the R language environment version 3.5.1 (The R Foundation for Statistical Computing, Vienna, Austria).

## Results

Primary tracheal tumors were diagnosed in 89 patients. Among them, 50 (56.2%) had SCC, 19 (21.3%) had ACC. The remaining histological diagnoses − 20 (22.5%) - were: non-small-cell carcinoma – 12 (13,5%), adenocarcinoma – 4 (4,5%), malignant peripheral nerve sheath tumor – 2 (2,2%), small-cell carcinoma – 1 (1,1%), unspecified carcinoma – 1 (1,1%). They were grouped for statistical purposes as ‘other’ and were not subsequently differentiated. Among the total study population, men slightly predominated (48 men and 41 women). The median age at diagnosis was 62 years (range: 51–68). The most common symptoms included dyspnea (37.1%) and hemoptysis (36.0%). In the group presented, 45 patients underwent primary radical treatment and 44 were eligible for exclusive palliative treatment. The distribution of demographic and clinical data in these two groups is summarized in Table 1.

**Table 1:** The distribution of demographic and clinical data by type of treatment

Clinicopathological Factors		Palliative treatment	Radical treatment	*p* ^1^
Number of patients		44	45	
Age—median (range)		65,50 (59,00–72,25)	56,00 (44,00–64,00)	0,001
Gender (%)	Female	13 (29,5)	28 (62,2)	0,004
	Male	31 (70,5)	17 (37,8)	
WHO performance status (%)	0	3 (6,8)	15 (35,7)	< 0,001
	1	13 (29,5)	24 (57,1)	
	2	20 (45,5)	3 (7,1)	
	3	8 (18,2)	0 (0,0)	
	No data	0 (0,0)	3 (6,7)	
Smoking status (%)	Never smoked	1 (2,3)	4 (8,9)	0,031
	Former smoker	3 (6,8)	8 (17,8)	
	Current smoker	19 (43,2)	8 (17,8)	
	No data	21 (47,7)	25 (55,6)	
Symptoms before diagnosis (%)	No symptoms	0 (0,0)	4 (8,9)	0,041
	Hoarseness	3 (6,8)	1 (2,2)	
	Dyspnea	20 (45,5)	13 (28,9)	
	Cough	4 (9,1)	3 (6,7)	
	Hemoptysis	14 (31,8)	18 (40,0)	
	Other	3 (6,8)	1 (2,2)	
	No data	0 (0,0)	5 (11,1)	
Lactate dehydrogenase (LDH)—median (range)		196,00 (158,00-229,00)	160,00 (138,00-169,50)	0,029
	No data	23 (52)	29 (64)	
Histology (%)	ACC	2 (4,5)	17 (37,8)	0,001
	Other	11 (25,0)	9 (20,0)	
	SCC	31 (70,5)	19 (42,2)	
Narrowing of the tracheal lumen (%)	≤ 49%	10 (22,7)	11 (24,4)	0,720
	≥ 50%	25 (56,8)	22 (48,9)	
	No data	9 (20,5)	12 (26,7)	
TNM				
T (%)	1	9 (28,1)	16 (40,0)	0,422
	2	23 (71,9)	24 (60,0)	
	No data	12 (27,3)	5 (11,1)	
N (%)	0	15 (45,5)	26 (68,4)	0,087
	1	18 (54,5)	12 (31,6)	
	No data	11 (25,0)	7 (15,6)	
M (%)	0	33 (75,0)	44 (97,8)	0,005
	1	11 (25,0)	1 (2,2)	
	No data	0 (0,0)	0 (0,0)	

^1^ To examine the significance of the association, Fisher’s exact test was used for categorical data and the Mann-Whitney U test was used for continuous data

ACC – adenoid cystic carcinoma; SCC – squamous-cell carcinoma

Surgical resection was performed in 13 patients (28.9%) out of 45 radically treated patients (10—ACC, 1—SCC, and 2: other histological type). Macroscopically and microscopically complete resection was performed in 1 patient diagnosed with ACC. Microscopically, tumor infiltration (R1 resection) was found in 8 patients with ACC, 1 with SCC, and 1 with a diagnosis of another histological type. Macroscopically, margin-positive resection (R2 surgery) was performed in 2 patients (1 – ACC, 1 – other). One patient with ACC developed a severe perioperative complication in the form of an episode of cervical spinal cord ischaemia. Postoperative radiation therapy (RT) was performed in 7 patients (6 - ACC, 1 - SCC) due to positive surgical margins (R1 surgery – 6 patients, R2 surgery – 1 patient). Treatment consisted of external beam RT with a total dose in the range of 60–70 Gy. Two patients received additional intratracheal radiation (7 and 14 Gy). Radiation therapy alone as the primary method of radical treatment was used in 25 patients (55.5%). The distribution among the histological types was as follows: 6—ACC, 15—SCC, 4—other. External beam RT was used in 15 patients with a total dose ≥ 60 Gy (range 60–70 Gy). In this group, 8 patients received intratracheal brachytherapy (range 6–14 Gy) to increase the total dose in combination with external beam irradiation. The remaining 6 patients received a dose of less than 60 Gy (range 40–58 Gy) and all of them received brachytherapy for dose escalation (range 5–22 Gy), 1 patient received brachytherapy alone with a dose of 36 Gy, and in 3 patients the radiation dose was not available in the documentation. Most of the patients treated with external beam RT received conventional fractionated RT (1 patient – hyperfractionation). Severe toxicities were reported in 8 patients. These were mainly acute esophageal reactions. Organ stenosis occurred in 3 patients. Radiochemotherapy (RTCT) as the initial radical treatment was used in 7 patients (1 – ACC, 3 – SCC, 3 – other). In 6 patients, treatment was administered sequentially, consisting of cisplatin and vinorelbine (1 patient received cisplatin and etoposide). Treatment typically consisted of 2 cycles of systemic treatment, followed by conventional conformal RT fractionated to a total dose of 60–66 Gy. In this group, 2 patients received intratracheal brachytherapy after external beam RT (60 Gy). One patient received concurrent treatment, including chemotherapy with carboplatin and paclitaxel, together with RT, at a total dose of 60 Gy. One patient received adjuvant RTCT after surgical treatment (R2 surgery). Table 2 lists the type of radical treatment and Table 3 shows the adjuvant treatment administered after resection. Radiotherapy was the most common type of treatment used in the group of patients treated with a palliative intention—33 patients (74.9%). The remaining patients were treated with chemotherapy (15.9%) or surgical palliative treatment to restore the airways (9.1%).

**Table 2:** Initial radical treatments of 45 patients with primary tracheal tumors

Radical treatment (*n* = 45)		
Modality	*n*	%
Surgery	13	28,9
Definitive radiation therapy (RT)	25	55,5
Definitive chemoradiation therapy (RTCT)	7	15,5

**Table 3:** Adjuvant therapy

Surgery (*n* = 13)			
Adjuvant therapy	Modality	*n*	%
No		5	38,5
Yes		8	61,6
	RT	7	53,9
	RTCT	1	7,7

RT, radiation therapy; RTCT, chemoradiation therapy

**Fig. 1:**
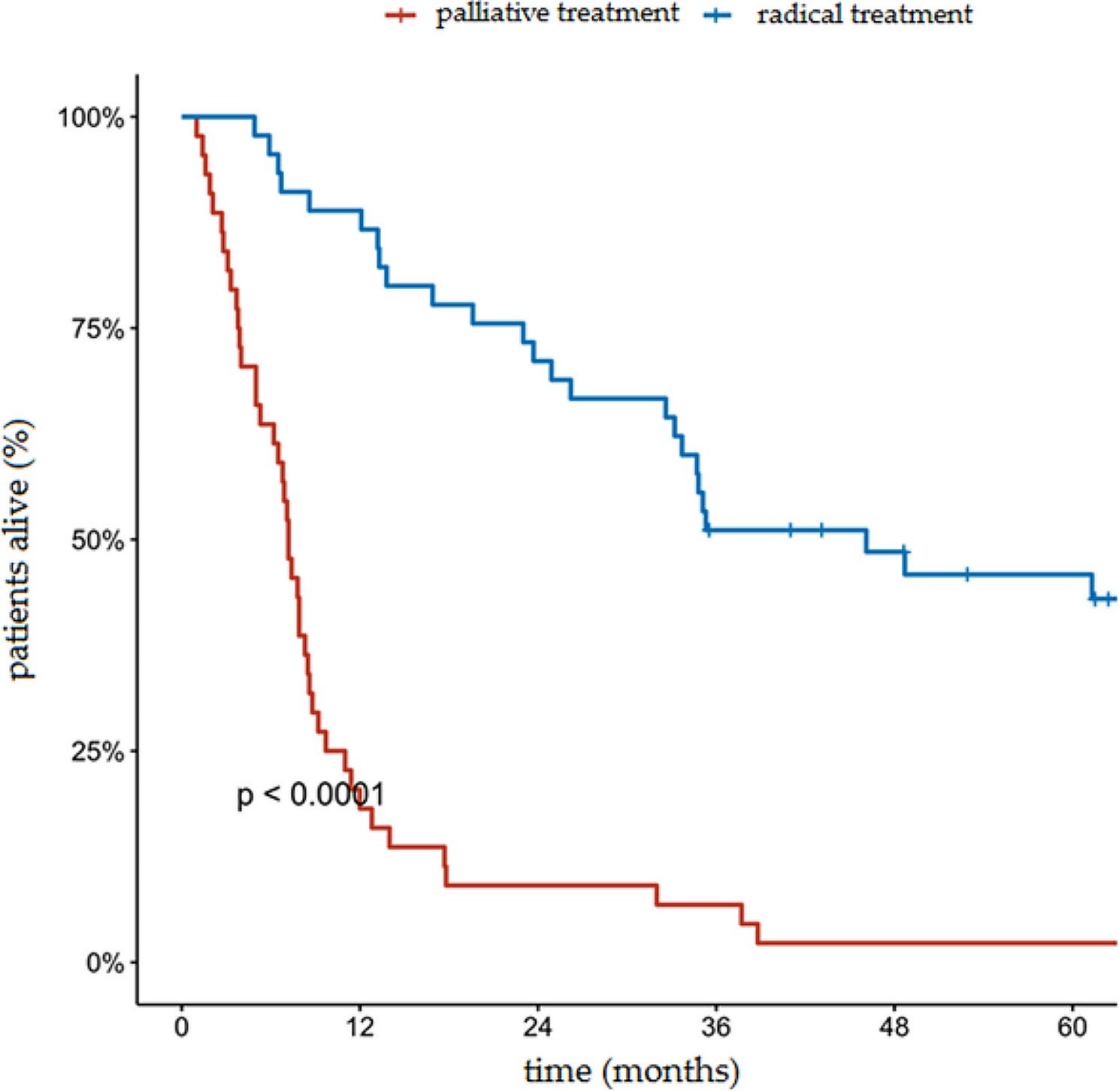
Cumulative probability of overall survival in the entire analyzed group of patients according to type of treatment (radical treatment vs. palliative treatment)

**Fig. 2:**
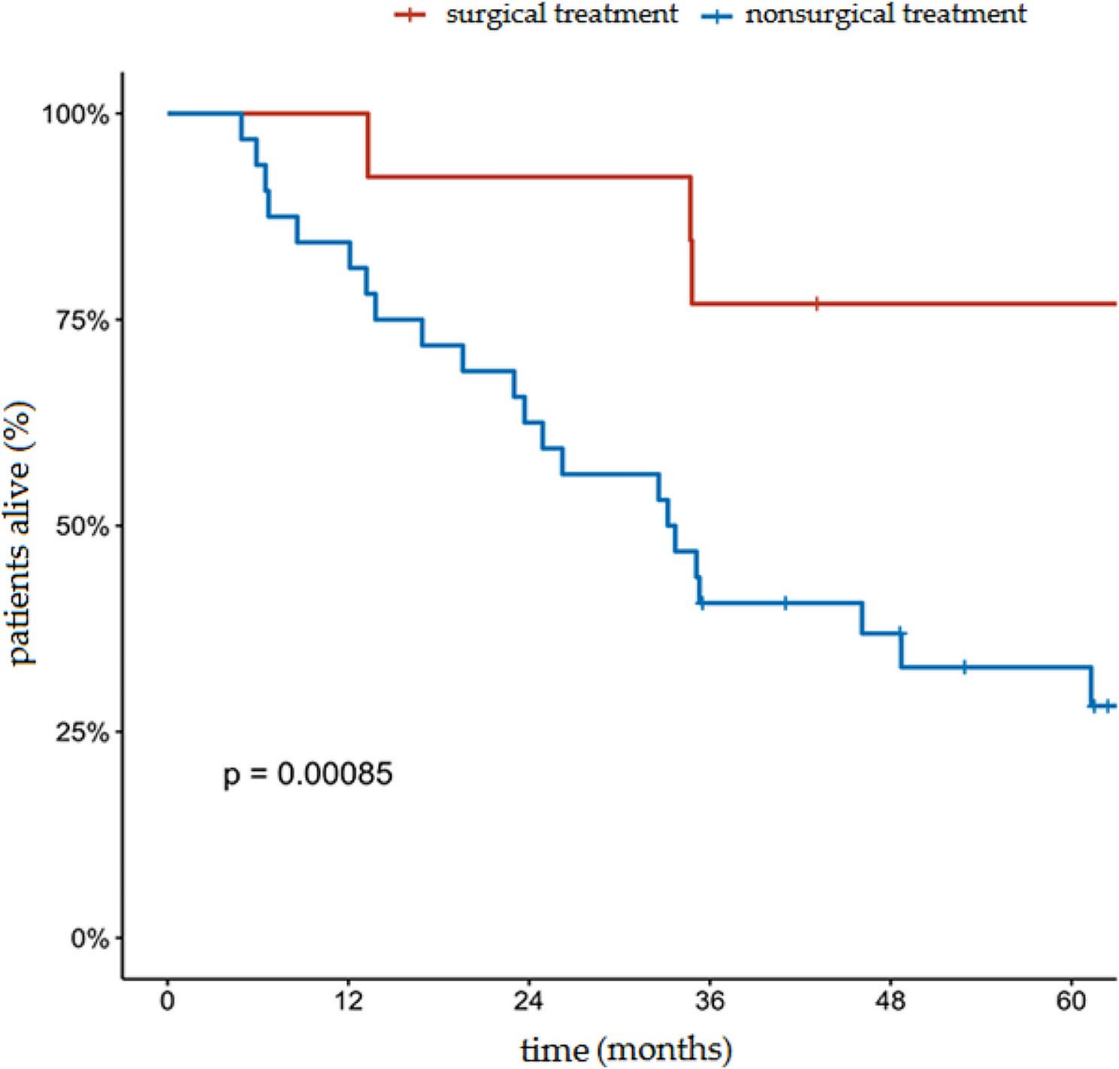
Cumulative probability of overall survival in patients receiving radical treatment according to the type of treatment (surgical treatment vs. non-surgical treatment)

**Fig. 3:**
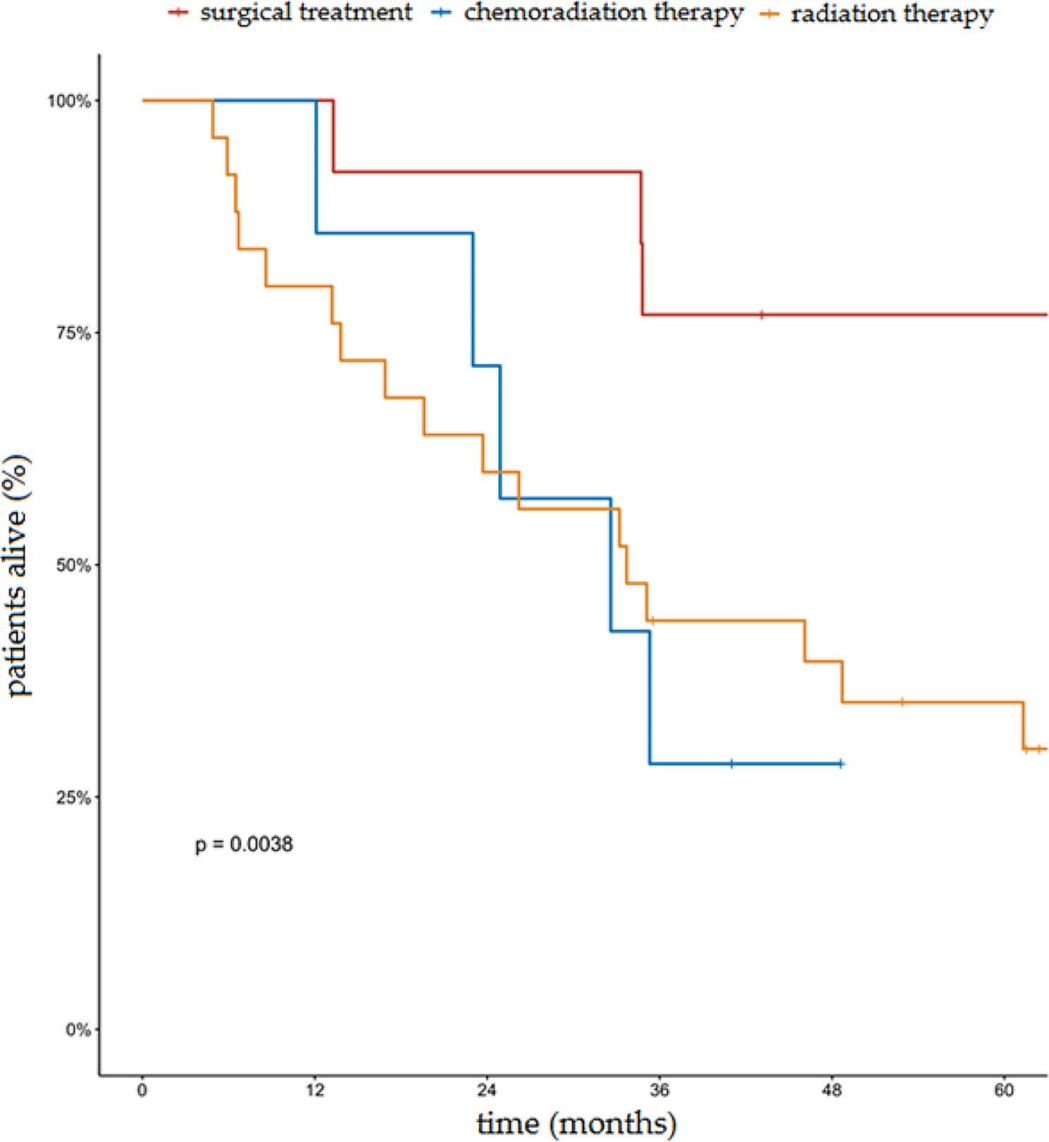
Cumulative probability of overall survival in patients receiving radical treatment according to the type of treatment (surgical treatment vs. radiation therapy and chemoradiation therapy)

The median OS in the analyzed group with the 95% confidence interval (CI) was 13.3 months (range: 9.2–26.2 months). The proportion of OS at 5 years in the entire analyzed group was 24.2% (95% CI, 16.7–35.2%). The 5 year OS rates in the group of patients who underwent radical treatment and in the group of patients who underwent palliative treatment was 45.9% and 2.3%, respectively (*p* < 0.001). The median OS in these two groups was 46.1 months and 7.2 months, respectively. Cumulative probability of overall survival in the entire group of patients analyzed according to the type of treatment is presented in Fig. 1. In the group of patients undergoing radical surgical treatment, the 5-year OS was 76.9% compared to 35.8% in the group of patients undergoing non-surgical treatment (Fig. 2). The 5- and 3-year OS of patients undergoing radical RT and RTCT were 35.2%, 0%, and 44.0%, 28.6%, respectively. Cumulative probability of OS in patients treated radically according to type of treatment is presented in Fig. 3.

Recurrence data were available for 42 patients in the radical treatment group. The median disease-free survival (DFS) in the analyzed group was 21.1 months (95% CI, 17.6–45.8 months). The 5-year DFS rate in the radically treated group was 25.4% (95% CI, 14.4–44.7%).

The median progression-free survival (PFS) in the palliative treatment group (44) was 3,95 months (95% CI, 2,9 − 6,3 months). The 5-year PFS rate in the palliative treated group was 2,3% (95% CI, 0,3–15,8%).

## Discussion

In the presented study, the 5-year OS rate in the group of patients undergoing radical treatment was 45.9%, while in the group of patients undergoing palliative treatment it was 2.3% (*p* < 0.0001). The median OS in these two groups was 46.1 months and 7.2 months, respectively. In the study by Makarewicz et al., which focused on irradiating patients with a diagnosis of tracheal cancer, the median OS was 26 months in the radical intention-treated group and 7.2 months in patients undergoing palliative RT [15].

In the group currently analyzed, 13 patients underwent radical surgical treatment. In this group, 5-year OS was 76.9% compared to 35.8% achieved in the group of patients treated with methods other than surgery. Radical surgical treatment has been documented to have value in the primary treatment of patients with tracheal cancer. Many studies have confirmed its superiority over other treatment methods. Patients who underwent surgical treatment had better OS with a median survival of 180 months compared to 36 months in the conservatively treated group (*p* < 0.001) [26]. In the next study, survival after diagnosis was significantly longer for patients undergoing curative intent resection, with a median overall survival of 120.3 month, as compared with 15.6 months for patients who underwent a debulking procedure and 24.5 months for patients who did not undergo surgery (*P* < 0.001) [29].

Direct comparison of therapeutic methods is difficult because most available data were obtained from a small series of retrospective observations. Comparative evaluation is complicated by the non-uniform selection of patients (patients in worse general condition and with more advanced disease are more frequently qualified for RT), the use of adjuvant RT, and different radiation techniques and doses. In a retrospective review of 308 cases of primary tracheal tumors from the Dutch Cancer Registry (1989–2002), the 5-year survival rate was 51% in surgically treated patients,, while among patients receiving RT alone, it was only 11% [3]. In the work of Licht and colleagues, among patients undergoing surgical treatment, radiation therapy, and radiation chemotherapy, the 5-year OS rates for these patient groups were 50%, 6%, and 0%, respectively [18]. In the study by Hetnal and his colleagues, the respective rates were 66%, 16%, and 0% [16]. Webb et al. presented similar results – better results were shown for patients treated surgically with adjuvant RT compared to patients treated with radiation with or without chemotherapy (*p* = 0.0003) [7]. However, the cited studies often included patients who received suboptimal radical treatment (palliative surgical procedures or lower radiation doses). In the current analysis, in the radically treated group, the 5-year OS for surgically treated patients was 76.9%, while among patients undergoing radical RT or RTCT, it was 35.2% and 0%, respectively (*p* = 0.0038). Recent data show that for 239 cases treated with surgery alone, the 5-year survival rate was 86.4%, and the 10-year survival rate was 55.6% [5]. For patients treated with surgery and postoperative radiotherapy, the 5-year and 10-year survival rates were 97.3% and 44.4%, respectively [5]. In cases treated with radiotherapy alone, the 5-year and 10-year survival rates were 34.9% and 16.1%, respectively [5]. Patients treated with a combination of surgery, radiotherapy, and chemotherapy had a 5-year survival rate of 88.9% [5].

In the previously cited study from 2019 [26] the authors reported that the use of radical surgical treatment was associated with better survival, with a median survival of 180 months compared to 48 months for RT (*p* < 0.001). There was no evidence that the use of adjuvant RT after surgical treatment significantly influenced these outcomes. The total radiation dose had an impact on survival in patients with SCC, with a median OS of 24 months when more than 60 Gy was used compared to 6 months for a lower dose. Several studies have delved into this topic in detail [14, 15, 22]. According to the authors of the meta-analysis, surgical treatment provides better results than RT for ACC and tracheal sarcomas. The role of radical RT compared to surgical treatment in patients with SCC requires further research [26].

About 30–50% of patients have unresectable lesions at the time of diagnosis [30]. For cases deemed unresectable or with contraindications to surgery, definitive radiotherapy is often proposed, showing a 5-year overall survival rate lower than that observed in surgically treated patients [31–33]. Despite the inferior survival rates with radiotherapy alone, it remains a valuable treatment option for unresectable tracheal cancers [5, 30, 32]. In radiotherapy, dosing is crucial for treatment outcomes. Mornex et al. suggested that the radiotherapy dose is a prognostic factor for primary tracheal tumors. The 5-year survival rate for patients who received more than 56 Gy of radiotherapy was 12%, while it dropped to 5% for those who received low-dose radiotherapy [22]. Levy et al. conducted a retrospective analysis of 31 cases of tracheal ACC treated at their center from 1984 to 2014. They found that whether the radiotherapy dose exceeded 60 Gy was an independent prognostic factor for progression-free survival [31]. Similar findings were reported by Licht et al., showing that tracheal malignancies treated with a radiation dose over 60 Gy had a 2-year survival rate superior to that of patients receiving less than 40 Gy [18]. High-dose radiotherapy may increase local tumor control rates and survival time. However, it also increases the risk of complications, such as tracheobronchial fistulas, airway stenosis, and tracheal necrosis [5, 14, 15]. With the advancement of radiotherapy techniques, complications after high-dose radiotherapy can be minimized. However, there is currently a lack of randomized trials on curative radiotherapy for primary tracheal tumors, and the optimal dose and fractionation of radiotherapy remain uncertain [5, 14, 15, 18, 22, 31].

The role of adjuvant radiotherapy remains controversial because of conflicting evidence. Achieving complete resection especially in cases of tracheal ACC is challenging, with success rates ranging from 42 to 57% [8, 31, 34]. Consequently, adjuvant radiotherapy is recommended for non-R0 resections [35–37], with doses typically ranging from 40 Gy to 77 Gy, and averaging at 55.8 Gy [5]. This therapy has been associated with improved survival and reduced risk of local recurrence and distant metastasis [7, 38–40]. Gaissert et al. reported positive survival outcomes in their cohort treated with adjuvant radiotherapy, highlighting its importance in improving prognosis [8]. Another study found that surgery combined with postoperative radiotherapy resulted in a 5-year survival rate of 97.3% and a 10-year survival rate of 44.4%, indicating the potential benefits of this treatment modality [5]. Furthermore, the SEER database analysis by Xie et al. revealed that adjuvant radiotherapy significantly improved overall survival and reduced mortality in tracheal cancer [41]. However, some studies, like those conducted by Yang et al. and Yusuf et al., showed no significant benefit from adjuvant radiotherapy for resected ACC and other tracheal cancers [39, 42]. Given the small number of patients, it was not possible to determine the value of adjuvant treatment in the current study.

Data from the literature suggest that more than half of patients with primary tracheal tumors could potentially be candidates for radical surgical treatment. However, due to the rare occurrence of tracheal tumors, lack of sufficient experience and often delayed diagnosis, suboptimal treatment is common [18, 43, 44]. A multidisciplinary audit of data from the Dutch Cancer Registry found that among 50 cases of locally advanced tracheal cancer, surgical treatment was applied in 24% of patients. Subsequent review identified 16 additional candidates who could have undergone surgical treatment, totaling 56% of the patients [44]. In our series, less than one third of the patients (28.9%) underwent radical surgical treatment. Due to the retrospective nature of our study, it cannot be concluded whether the remaining patients received suboptimal treatment. The additional factors could influence the abandonment of surgery, but were not documented. However, the above data should serve as a basis for a careful assessment of indications and contraindications for resection to ensure the most optimal treatment. For this reason, treatment should be performed in specialized centers, as it usually requires the collaboration of a multidisciplinary team experienced in the treatment of these rare tumors. This may lead to the qualification of a greater number of patients for surgical resection and the potential for a better prognosis.

In the management of tracheal cancer, the choice between surgical resection and other treatments such as radiation reflects a complex interplay of patient characteristics and institutional factors. Studies indicate that patients undergoing resection are generally younger, with a lower Charlson Comorbidity Index, and more likely to be insured privately. These patients typically present with ACC, lower-grade, and smaller-sized tumors. Notably, resections are predominantly performed at academic or research centers, underscoring the specialization required for such procedures [45]. For instance, at Massachusetts General Hospital, a remarkable 82% resection rate has been reported, particularly among ACC patients, which is considerably higher than the rates observed in other studies [3, 8, 18]. Among the 1379 patients with tracheal cancer identified in a recent study, 25% underwent surgical resection. Factors positively influencing the likelihood of undergoing resection included not only younger age and tumor characteristics but also higher education levels and treatment at academic institutions. Interestingly, patients traveling more than 45 km to receive treatment were also more likely to undergo surgery [29]. The likelihood of receiving surgical resection or curative radiation decreases with age, with younger patients being the most likely to receive these interventions. In contrast, older patients more often receive palliative radiotherapy or no therapy at all, highlighting age as a critical determinant of treatment modality [33].

Initially, palliative treatment was administered to 44 patients. All patients presented symptoms, with the most common being dyspnea (45.5%) and hemoptysis (31.8%). Most of the patients had a WHO performance status of 2 or 3 (63.7%). Distant metastases were present at the time of diagnosis in 11 patients. Most patients received palliative RT (*n* = 33), while others received chemotherapy (*n* = 7) or palliative surgical procedures performed due to symptoms caused by tracheal stenosis in patients not eligible for other palliative treatment methods (*n* = 4). In the current study, an attempt was made to analyze the impact of the specific palliative treatment method (radiation therapy/chemotherapy) on patient outcomes. There was no statistically significant impact of the type of treatment on PFS (*p* = 0.57) and OS (*p* = 0.68) in the palliative treatment group of patients. Given the sample size, there are doubts about whether the results obtained can be extrapolated to the entire population.

Relevant studies specifically addressing exclusive palliative treatment for patients diagnosed with tracheal tumors appear to be lacking. In most studies, patients receiving palliative treatment were part of the overall analyzed group. In studies involving palliative RT, the authors observed a good palliative effect in terms of relieving obstruction and reducing the severity of symptoms [15, 16, 22, 30]. The percentages of patients who showed improvement in the observation after 3 months from completion of RT were 56.3% for dyspnea and 72.2% for hemoptysis, with an average response duration of 12.5 months [15]. For some patient who present with metastatic disease, also palliative resection can be used to relieve airway obstruction in cases where a tracheostomy is not feasible [8, 46]. The efficacy and safety of palliative surgical procedures for patients with obstruction due to ACC was presented in the work of Lee et al. [24]. Endobronchial treatment is palliative, but it may be assumed that it also can prolong life [33]. It should, in addition to surgery, medication and radiotherapy, be available in any combination for these patients [33] but the prognosis for patients who cannot undergo radical treatment is generally poor.

The presented work has several limitations. Although this article includes one of the larger series of patients with primary tracheal tumors, the number of patients is still small. Another limitation in the interpretation of the results of this retrospective study is limited by the selection of treatment methods, with surgical resection performed much more frequently in cases of ACC (ACC – 52.6% vs. SCC – 2%). The data presented align with those available in the literature, especially in terms of a better prognosis for patients diagnosed with ACC. Another challenge is the lack of a unified TNM classification system for this group of tumors, making it difficult to perform analyses and compare study results.

## Conclusions

An optimal method of radical treatment for patients with primary tracheal tumors is resection. Efforts should be made to improve and accelerate the diagnosis of patients with a suspected primary tracheal tumor and to refer patients to reference centers specialized in the management of these rare tumors. Treatment options, including in-depth consideration of surgical treatment options, should be decided by a multidisciplinary team.

## Data Availability

Data may be available upon reasonable request from the corresponding author and with permission of the National Research Institute of Oncology, Warsaw, Poland.
